# Early Small Airway Mechanics and Functional Correlates in Asymptomatic Smokers: An Impulse Oscillometry-Based Study

**DOI:** 10.1007/s00408-026-00902-1

**Published:** 2026-06-10

**Authors:** Alperen Aksakal, Buğra Kerget, Ayşenur Sağ, Mustafa Buğra Coşkuner

**Affiliations:** 1 Department of Pulmonary Diseases, Ataturk University School of Medicine, 25240 Yakutiye, Erzurum, Turkey; 2 Department of Pulmonary Diseases, Aksaray University School of Medicine, 68200 Aksaray, Turkey

**Keywords:** Impulse oscillometry, Small airway dysfunction, Smoking, Exercise capacity, Muscle oxygenation, Spirometry

## Abstract

**Background:**

Early mechanical alterations in small airways may occur in asymptomatic smokers despite preserved spirometric lung function. This study aimed to evaluate respiratory system mechanics using impulse oscillometry (IOS) in asymptomatic smokers and to investigate their relationship with exercise capacity and muscle oxygenation.

**Methods:**

In this cross-sectional study, university students who were current smokers and never-smokers were included. All participants underwent spirometry and IOS measurements before and after the six-minute walk test (6MWT). IOS parameters included resistance (R5–R25, R5–R20) and reactance (X5, X5VEX, X5VIN, AX, Fres). Respiratory muscle strength (MIP/MEP) and muscle oxygen saturation (SmO₂) measured by near-infrared spectroscopy at the vastus lateralis were also assessed. Associations between IOS parameters, smoking exposure, exercise capacity, and muscle oxygenation were analyzed.

**Results:**

Despite comparable FVC % predicted, FEV₁ % predicted, and FEV₁/FVC values, smokers demonstrated higher resistance indices (R5, R5–R20) as well as higher AX and Fres values, and lower reactance parameters (X5, X5VIN) compared with non-smokers (p < 0.001 for most comparisons). Smoking exposure correlated positively with resistance parameters and negatively with X5. Smokers exhibited reduced 6MWT distance and lower SmO₂ levels. In multivariable analysis, lower R5 and higher X5VIN were associated with longer 6MWT distance (p = 0.008 and p < 0.001, respectively).

**Conclusions:**

IOS can detect early small airway mechanical alterations in asymptomatic smokers despite preserved spirometry. The associations of these changes with exercise capacity and muscle oxygenation suggest that early small airway involvement may have functional correlates. IOS may serve as a complementary tool for the multidimensional assessment of smoking-related airway effects.

**Supplementary Information:**

The online version contains supplementary material available at 10.1007/s00408-026-00902-1.

## Introduction

Smoking is associated with structural and mechanical alterations, particularly in the small airways (< 2 mm), before the onset of clinically apparent symptoms [1]. Inflammation and luminal narrowing in this region are often not detected by conventional spirometry due to their limited contribution to total airway resistance; this suggests that ventilation heterogeneity and impaired elastic properties may develop in the peripheral airways even when spirometric measures remain within normal limits [2, 3].

Respiratory oscillometry, including forced oscillation technique (FOT) and impulse oscillometry (IOS), is an effort-independent functional measurement approach that evaluates respiratory system impedance through the application of small pressure oscillations to the airway system during tidal breathing [4, 5]. In IOS, which was used in the present study, resistance and reactance parameters reflect different components of respiratory system mechanics and allow the evaluation of functional changes, particularly in the peripheral airways. Because low-frequency oscillations can be transmitted to the distal airways, resistance measured at 5 Hz (R5) represents total airway resistance, whereas resistance at 20 Hz (R20) predominantly reflects central airway resistance. The difference between R5 and R20 (R5–R20) is commonly considered to reflect frequency-dependent resistance and ventilation heterogeneity, although it should not be interpreted as a direct measure of a single anatomical airway region [5, 6]. Similarly, reactance parameters, including X5, the reactance area (AX), and the resonant frequency (Fres), provide information on the elastic properties of the peripheral airways and ventilation heterogeneity [6, 7]. IOS has been shown to be a complementary technique capable of detecting early changes in small airway function, even in individuals with normal conventional spirometry [6, 7].

In recent years, IOS has been increasingly used to assess small airway function in asthma, chronic obstructive pulmonary disease, and other respiratory diseases [8, 9]. Several studies have demonstrated that IOS parameters are particularly sensitive to mechanical alterations in the peripheral airways and may provide complementary information for the early detection of small airway dysfunction [9, 10]. Furthermore, even in smokers with normal spirometric lung function, IOS parameters have been shown to indicate early small airway dysfunction [3]. FOT-based studies have also shown that smoking-related changes in respiratory mechanics can be detected early and may provide information complementary to conventional spirometric assessment [11–14]. In asymptomatic smokers, distal airway dysfunction assessed by FOT has been associated with pulmonary inflammation, supporting the physiological relevance of oscillometric abnormalities in this population [15]. However, the relationship between these changes and functional outcomes has been insufficiently investigated, particularly in young and asymptomatic smokers. Moreover, ventilatory mechanical responses during exercise have not been sufficiently characterized in conjunction with IOS parameters, and their association with functional capacity has not been sufficiently defined.

In this study, small airway function was assessed using IOS in university student smokers and non-smokers, and the association of IOS parameters measured before and after exercise with functional capacity and muscle oxygenation was investigated. We hypothesized that asymptomatic smokers without spirometric impairment would demonstrate measurable differences in IOS-derived respiratory system mechanics, with these differences being associated with smoking exposure, exercise capacity, and muscle oxygenation.

## Methods

### Study Design and Participants

This observational, cross-sectional study was conducted among university students who were smokers and non-smokers at Atatürk University. Data were collected between June 2025 and January 2026.

Active smokers and never-smokers were included in the study. Current smoking was defined as the use of conventional tobacco-based cigarettes, and smoking exposure was recorded in pack-years. No participants reported using electronic cigarettes, vaping products, or non-tobacco nicotine products. Participants with known chronic lung disease, cardiovascular disease, or systemic conditions that could affect exercise capacity were excluded. Participants with active respiratory tract infections, clinical findings suggestive of active COVID-19, or persistent respiratory or physical symptoms suggestive of post-COVID sequelae were also excluded. Participants with physical limitations that could interfere with test performance or those engaged in regular intense physical activity were also excluded.

Written informed consent was obtained from all participants. The study was conducted in accordance with the Declaration of Helsinki and was approved by the Atatürk University Clinical Research Ethics Committee (Decision No: B.30.2.ATA.0.01.00/961).

### Study Protocol

All measurements were conducted on the same day under similar environmental conditions. Before testing, participants were instructed to avoid strenuous exercise, heavy meals, and clothing that could restrict movement.

First, exhaled carbon monoxide (CO) levels were measured in all participants. Pulmonary function testing was then performed, with IOS conducted before spirometry for each participant. Respiratory muscle strength was evaluated using maximal inspiratory pressure (MIP) and maximal expiratory pressure (MEP).

A near-infrared spectroscopy (NIRS) device was placed over the vastus lateralis muscle to monitor muscle oxygenation before exercise. All participants completed a six-minute walk test (6MWT).

Following exercise, IOS measurements, along with MIP and MEP assessments, were repeated. Muscle oxygenation was continuously recorded throughout the exercise period.

### Pulmonary Function Tests

Pulmonary function tests were performed in all participants in accordance with the American Thoracic Society/European Respiratory Society (ATS/ERS) recommendations [16]. Spirometry was performed by the same technician in accordance with ATS/ERS standards, and the best values from at least three acceptable maneuvers were used for analysis.

IOS measurements were performed using the MasterScreen IOS system (Vyaire Medical GmbH, Hoechberg, Germany). Measurements were obtained during tidal breathing in a seated position with a nose clip. The device was calibrated daily, and only technically acceptable measurements were included in the analysis [5].

During IOS testing, pressure oscillations were applied over a frequency range of 5–35 Hz, and recordings of at least 30 s were obtained. The mean of at least three acceptable measurements was used. Resistance parameters (R5, R10, R15, R20, and R25), as well as the R5–R20 difference, were analyzed. Reactance parameters included X5, expiratory reactance (X5VEX), and inspiratory reactance (X5VIN), along with AX and Fres. Z5, reflecting respiratory system impedance, was also included in the analysis [6].

### Respiratory Muscle Strength

Respiratory muscle strength was assessed using MIP and MEP measurements with a portable, validated device (RP Check MIP & MEP & SNIP; MediTrack, Belgium). Measurements were performed with participants in an upright seated position using a nose clip. MIP and MEP measurements were conducted in accordance with ATS/ERS recommendations, and the highest value from at least three acceptable maneuvers was used for analysis [17]. MIP and MEP values were expressed as percentage of predicted values (% predicted) according to the reference equations integrated into the device software.

### Six-Minute Walk Test

The 6MWT was performed in accordance with ATS recommendations [18]. The test was conducted in a 30-m straight corridor. Participants were allowed to rest for at least 15 min before the test, and baseline parameters were recorded. Participants were instructed to walk as far as possible at a self-selected pace. Participants were monitored for symptoms during the test, and the test was terminated if necessary. The total distance walked was recorded at the end of the test.

### Exhaled CO and Smoking Index (HSI)

Exhaled CO levels were measured using a Pico™ Smokerlyzer® device (Bedfont Scientific Ltd., Kent, UK). Participants were instructed to perform a deep inspiration, hold their breath for approximately 15 s, and then exhale slowly and completely. Measurements were performed in accordance with a standardized protocol.

Smoking exposure was assessed in pack-years. Smoking intensity was evaluated using the heaviness of smoking index (HSI). The HSI score was calculated based on the time to first cigarette and the number of cigarettes smoked per day [19].

### Muscle Oxygenation

Muscle oxygenation was assessed using near-infrared spectroscopy (NIRS) with a MOXY monitor (MOXY®, Minnesota, USA). The device was placed over the vastus lateralis muscle. Measurements were initiated at the beginning of the 6MWT and recorded continuously throughout the test. Muscle oxygenation was expressed as muscle oxygen saturation (SmO₂, %), defined as the ratio of oxygenated hemoglobin to total hemoglobin. For analysis, values obtained at the beginning and at the end of the 6MWT were used.

### Statistical Analysis

The required sample size was calculated a priori using G*Power software version 3.1.9.7 (Heinrich Heine University, Düsseldorf, Germany). The calculation was based on the difference in R5–R20 values between asymptomatic smokers and non-smokers reported by Pisi et al. [3]. Assuming a power of 90%, a one-sided α error probability of 0.05, and a 1:1 allocation ratio, the minimum required sample size was calculated as 38 participants per group. Accordingly, 40 smokers and 40 non-smokers were included in the study.

All statistical analyses were conducted using IBM SPSS Statistics version 27.0 (IBM Corp., Armonk, NY, USA). The distribution of continuous variables was evaluated using the Shapiro–Wilk test. As most variables were not normally distributed, results are presented as median (IQR).

Comparisons between smokers and non-smokers were performed using the Mann–Whitney U test for continuous variables. Categorical variables were compared using the chi-square test or Fisher’s exact test when appropriate. Changes in IOS parameters, respiratory muscle strength, and muscle oxygenation before and after the 6MWT were analyzed using the Wilcoxon signed-rank test within each group.

The relationships between smoking exposure (pack-years), baseline SmO₂ levels, and IOS parameters were evaluated using Spearman’s correlation analysis. Receiver operating characteristic (ROC) analysis was performed to evaluate the discriminatory performance of pre-exercise IOS parameters for identifying lower baseline SmO₂ levels (< 80%). Area under the curve (AUC) values with 95% confidence intervals were calculated. A multivariable linear regression model was constructed to determine independent predictors of 6MWT distance in smokers. Variables included in the model were pulmonary function parameters, IOS indices, respiratory muscle strength, and oxygenation variables.

Multicollinearity was assessed using the variance inflation factor (VIF) and tolerance values, and no significant multicollinearity was detected. All statistical tests were two-sided, and a p-value < 0.05 was considered statistically significant.

## Results

The median (IQR) age of participants was 25 (24–31), with no significant difference between smokers and non-smokers (25 [24–27] vs. 25 [24–26.5], respectively; p = 0.64). A total of 47.5% of participants were female, and sex distribution was similar between the groups (p = 0.65). The median (IQR) smoking exposure in the smoking group was 18 (15–20) pack-years.

Demographic characteristics, pre-6MWT exhaled CO levels, pulmonary function test results, IOS parameters, as well as MIP, MEP, SmO₂, and 6MWT distance are presented in Table 1. Exhaled CO levels and all resistance parameters (R5, R10, R15, R20, R25, and R5–R20), as well as AX, Fres, and Z5 values, were significantly higher in smokers (p < 0.001 for all comparisons). In contrast, FVC (L) and FEV₁ (L) values, reactance parameters (X5, X5VEX, and X5VIN), 6MWT distance, and pre-exercise SmO₂ levels were higher in non-smokers (p = 0.008, p = 0.008, p < 0.001, p < 0.001, p < 0.001, p = 0.002, and p < 0.001, respectively). Despite these differences in absolute volumes, no significant between-group differences were observed in FVC % predicted, FEV₁ % predicted, or FEV₁/FVC values.

**Table 1:** Demographic characteristics, pre-6MWT exhaled CO levels, pulmonary function and IOS parameters, respiratory muscle strength, oxygen saturation, muscle oxygenation, and 6MWT distance in smokers and non-smokers

	Non-smokers n = 40 Median (IQR)	Smokers n = 40 Median (IQR)	p
Age (years)	25.0 (24.0–26.5)	25.0 (24.0–27.0)	0.64
Height (cm)	180.0 (170.0–180.0)	177.0 (174.0–182.0)	0.07
Weight (kg)	82.0 (72.0–88.0)	80.0 (76.0–87.0)	0.06
BMI (kg/m^2^)	26.0 (25.0–28.0)	27.0 (25.0–28.0)	0.21
CO (ppm)	2.0 (1.0–2.0)	16.0 (13.0–22.0)	< 0.001
CO (%)	1.27 (1.11–2.55)	1.91 (1.43–2.95)	0.002
FVC (L)	5.2 (4.26–6.17)	4.41 (3.91–5.47)	0.008
FVC (%)	112.0 (96.0–115.0)	106.0 (100.0–117.0)	0.89
FEV_1_ (L)	4.54 (4.27–5.03)	3.8 (3.29–4.53)	0.008
FEV_1_ (%)	106.0 (97.0–113.0)	102.0 (96.5–110.5)	0.31
FEV_1_/FVC (%)	82.3 (80.7–86.9)	82.4 (78.5–85.9)	0.65
R5 (kPa/(L/s))	0.25 (0.24–0.29)	0.36 (0.30–0.38)	< 0.001
R10 (kPa/(L/s))	0.23 (0.21–0.27)	0.30 (0.27–0.34)	< 0.001
R15 (kPa/(L/s))	0.22 (0.19–0.24)	0.30 (0.25–0.32)	< 0.001
R20 (kPa/(L/s))	0.24 (0.19–0.25)	0.30 (0.23–0.32)	< 0.001
R25 (kPa/(L/s))	0.24 (0.19–0.27)	0.31 (0.22–0.34)	< 0.001
R5–R20 (kPa/(L/s))	0.03 (0.01–0.055)	0.07 (0.05–0.07)	< 0.001
AX (kPa/L)	0.26 (0.15–0.405)	0.57 (0.40–0.91)	< 0.001
X5 (kPa/(L/s))	-0.06 (-0.08–-0.04)	-0.09 (-0.11–-0.08)	< 0.001
X5VEX (kPa/(L/s))	-0.06 (-0.08–-0.04)	-0.10 (-0.12–-0.08)	< 0.001
X5VIN (kPa/(L/s))	-0.05 (-0.08–0.00)	-0.09 (-0.10–-0.05)	< 0.001
Fres (Hz)	12.50 (11.58–13.01)	18.25 (16.52–25.52)	< 0.001
Z5 (kPa/(L/s))	0.26 (0.25–0.29)	0.37 (0.32–0.39)	< 0.001
6MWT distance (m)	605.0 (558.0–637.5)	564.0 (534.0–582.0)	0.002
MIP (% predicted)	71.0 (55.0–96.0)	63.0 (53.0–84.0)	0.29
MEP (% predicted)	67.0 (53.0–81.0)	65.0 (57.0–78.0)	0.27
SpO_2_ (%)	96.0 (96.0–98.0)	96.0 (95.0–97.0)	0.24
SmO₂ (%)	84.0 (80.0–86.0)	75.0 (69.0–82.0)	< 0.001
HSI	NA	3.0 (2.0–4.0)	NA
Cigarette (pack/year)	NA	18.0 (15.0–20.0)	NA

6MWT: six-minute walk test; AX: reactance area; BMI: body mass index; CO: carbon monoxide; FEV₁: forced expiratory volume in 1 s; Fres: resonant frequency; FVC: forced vital capacity; HSI: heaviness of smoking index; IOS: impulse oscillometry; IQR: interquartile range; MIP/MEP: maximal inspiratory/expiratory pressure; R5–R25: resistance at 5–25 Hz; SmO₂: muscle oxygen saturation; SpO₂: peripheral oxygen saturation; X5: reactance at 5 Hz; X5VEX/X5VIN: expiratory/inspiratory reactance; Z5: respiratory system impedance at 5 Hz

IOS parameters, along with MIP, MEP, and SmO₂ levels obtained after the 6MWT, are presented in Table 2. In the post-exercise assessment, all resistance parameters (R5, R10, R15, R20, R25, and R5–R20), as well as AX, Fres, and Z5 values, were significantly higher in smokers than in non-smokers (p < 0.05 for all comparisons). In contrast, reactance parameters (X5, X5VEX, and X5VIN) and SmO₂ levels were higher in non-smokers (p = 0.002, p = 0.01, p = 0.04, and p < 0.001, respectively).

**Table 2:** Post-6MWT IOS parameters, respiratory muscle strength, and muscle oxygenation in smokers and non-smokers

	Non-smokers n = 40	Smokers n = 40	p
R5 (kPa/(L/s))	0.26 (0.23–0.30)	0.37 (0.27–0.40)	< 0.001
R10 (kPa/(L/s))	0.23 (0.21–0.26)	0.30 (0.22–0.33)	< 0.001
R15 (kPa/(L/s))	0.22 (0.20–0.25)	0.28 (0.23–0.31)	< 0.001
R20 (kPa/(L/s))	0.23 (0.20–0.25)	0.26 (0.24–0.32)	< 0.001
R25 (kPa/(L/s))	0.24 (0.21–0.28)	0.27 (0.25–0.35)	0.002
R5–R20 (kPa/(L/s))	0.04 (0.02–0.05)	0.06 (0.04–0.08)	0.001
AX (kPa/L)	0.30 (0.17–0.44)	0.49 (0.34–0.73)	< 0.001
X5 (kPa/(L/s))	-0.07 (-0.08–-0.04)	-0.09 (-0.12–-0.06)	0.002
X5VEX (kPa/(L/s))	-0.08 (-0.09–-0.04)	-0.09 (-0.11–-0.08)	0.01
X5VIN (kPa/(L/s))	-0.06 (-0.08–-0.04)	-0.08 (-0.13–-0.06)	0.04
Fres (Hz)	14.8 (11.41–17.03)	16.4 (12.61–23.99)	0.04
Z5 (kPa/(L/s))	0.28 (0.24–0.30)	0.38 (0.28–0.40)	< 0.001
MIP (% predicted)	76 (49–97)	69 (58–89)	0.58
MEP (% predicted)	68 (63–75)	67 (63–78)	0.7
SmO₂ (%)	82 (75–85)	70 (60–74)	< 0.001

AX: reactance area; Fres: resonant frequency; IOS: impulse oscillometry; MIP/MEP: maximal inspiratory/expiratory pressure; R5–R25: resistance at 5–25 Hz; SmO₂: muscle oxygen saturation; X5: reactance at 5 Hz; X5VEX/X5VIN: expiratory/inspiratory reactance; Z5: respiratory system impedance at 5 Hz

**Fig. 1:**
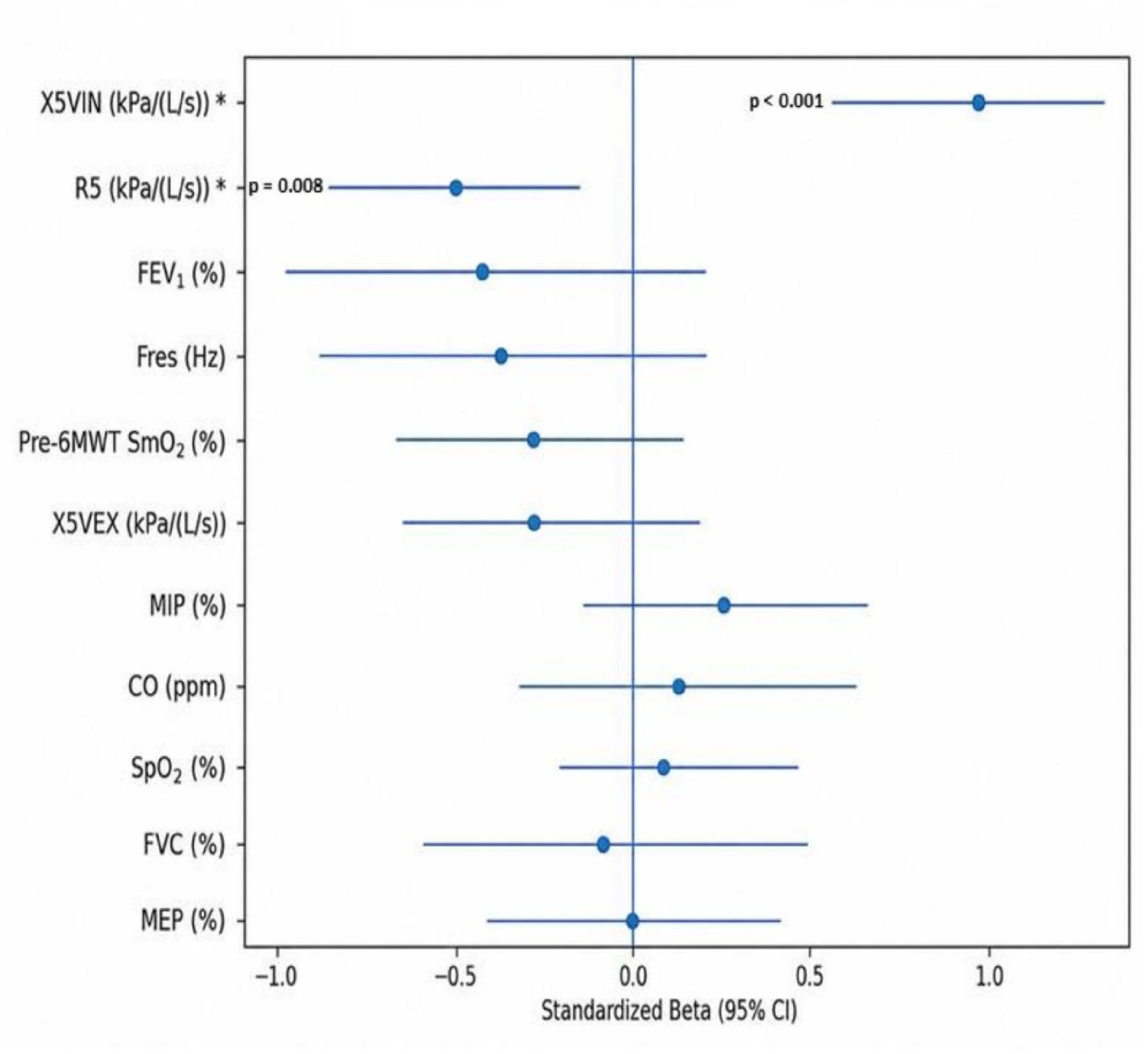
Multivariable regression analysis of factors associated with 6MWT distance in smokers

The results of the multivariable regression analysis evaluating factors associated with 6MWT distance in smokers are presented in Table 3 and Fig. 1. In this analysis, lower pre-6MWT R5 levels (B = -347.95, β = -0.471, p = 0.008) and higher X5VIN levels (B = 901.719, β = 0.836, p < 0.001) were associated with longer 6MWT distance.

**Table 3:** Multivariable regression analysis of factors associated with 6MWT distance in smokers

	Unstandardized Coefficients		Standardized Coefficients	t	p-value	Collinearity Statistics	
	B	Std. Error	β			Tolerance	VIF
(Constant)	595.796	848.148		0.702	0.488		
CO (ppm)	0.874	1.9	0.112	0.46	0.649	0.287	3.479
FVC (%)	-0.172	1.137	-0.04	-0.152	0.881	0.244	4.097
FEV_1_ (%)	-1.567	1.098	-0.407	-1.427	0.164	0.21	4.758
R5 (kPa/(L/s))	-347.95	122.231	-0.471	-2.847	0.008	0.625	1.6
X5VEX (kPa/(L/s))	-301.699	259.557	-0.218	-1.162	0.255	0.489	2.046
X5VIN (kPa/(L/s))	901.719	222.482	0.836	4.053	< 0.001	0.403	2.483
Fres (Hz)	-3.032	2.303	-0.331	-1.316	0.198	0.271	3.695
MIP (% predicted)	0.264	0.344	0.159	0.769	0.448	0.402	2.487
MEP (% predicted)	-0.029	0.462	-0.013	-0.064	0.95	0.404	2.478
SpO_2_ (%)	4.361	8.985	0.086	0.485	0.631	0.54	1.852
Pre-6MWT SmO₂ (%)	-1.048	0.805	-0.236	-1.302	0.203	0.523	1.913

6MWT: six-minute walk test; CO: carbon monoxide; FEV₁: forced expiratory volume in 1 s; Fres: resonant frequency; FVC: forced vital capacity; MIP/MEP: maximal inspiratory/expiratory pressure; R5: resistance at 5 Hz; SmO₂: muscle oxygen saturation; SpO₂: peripheral oxygen saturation; X5VEX/X5VIN: expiratory/inspiratory reactance

**Fig. 2:**
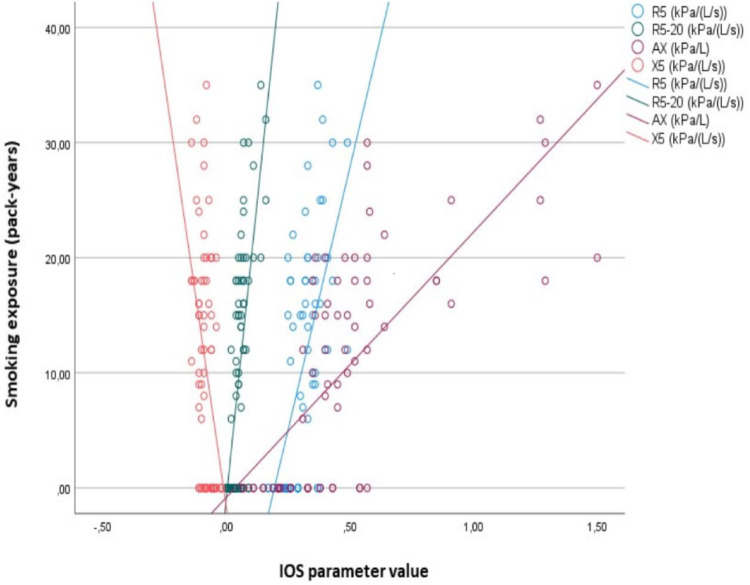
Correlations between cigarette pack-years and pre-6MWT IOS parameters

The correlation analysis between smoking exposure and pre-6MWT IOS parameters is presented in Fig. 2. Significant positive correlations were observed between smoking exposure (pack-years) and resistance parameters (R5: r = 0.669, p < 0.001; R5–R20: r = 0.686, p < 0.001; AX: r = 0.767, p < 0.001). In contrast, a significant negative correlation was observed between X5 levels and smoking exposure (r =  − 0.416, p < 0.001).

Correlation analysis demonstrated significant associations between pre-6MWT IOS parameters and baseline SmO₂ levels (Supplementary Table 1). Baseline SmO₂ showed negative correlations with resistance-related parameters, including R5 (r =  − 0.272, p = 0.021), R5–R20 (r =  − 0.340, p = 0.003), AX (r =  − 0.424, p < 0.001), and Fres (r =  − 0.398, p = 0.001). In contrast, positive correlations were observed with reactance parameters, including X5 (r = 0.244, p = 0.039) and X5VEX (r = 0.269, p = 0.023). No significant correlation was found between X5VIN and baseline SmO₂ (p = 0.243).

**Fig. 3:**
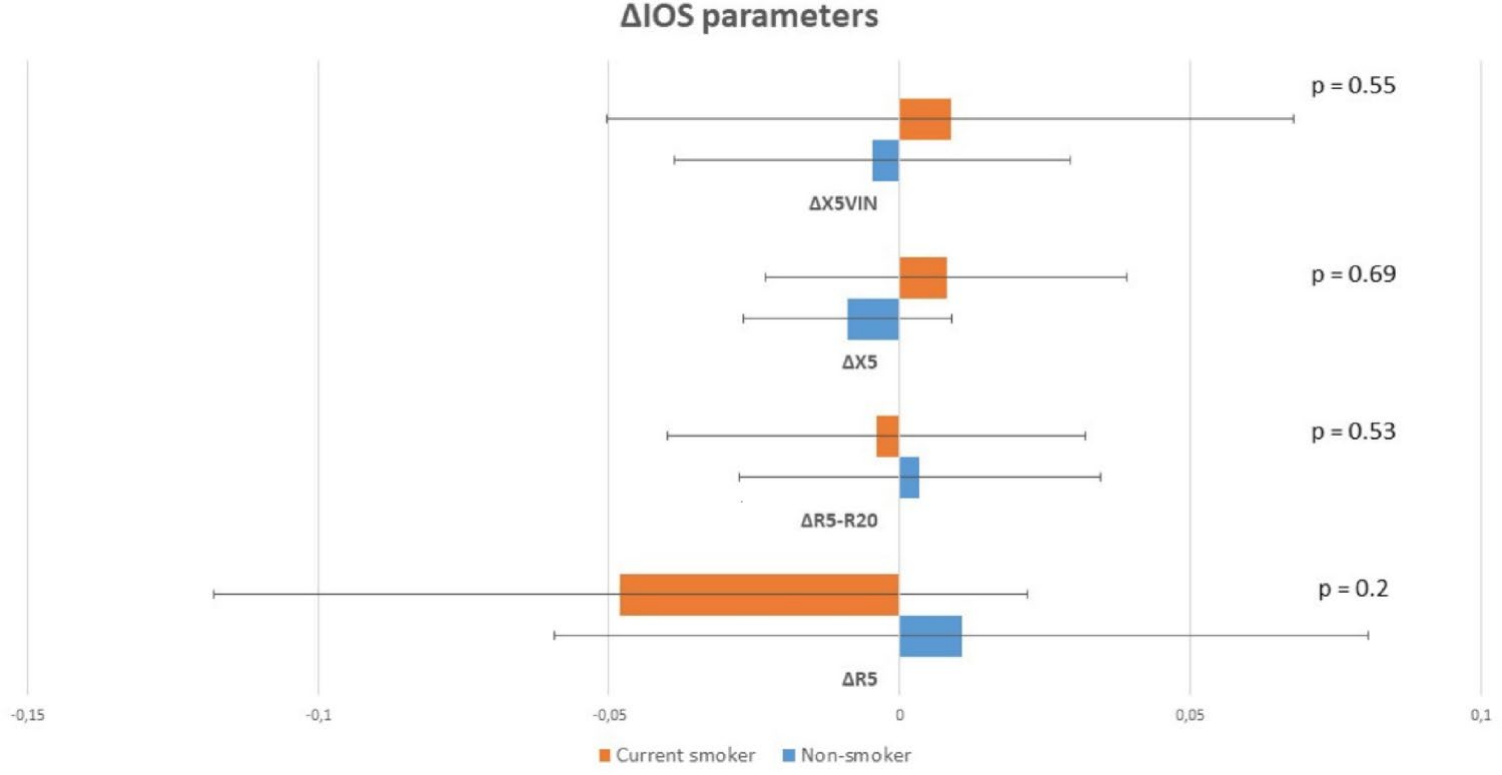
Changes in IOS parameters measured before and after the 6MWT in smokers and non-smokers

The comparison of changes in IOS parameters before and after the 6MWT between smokers and non-smokers is presented in Fig. 3. In the post-exercise assessment, a decreasing trend was observed in resistance parameters (R5 and R5–R20) and an increasing trend in reactance parameters (X5 and X5VIN) in smokers. However, no statistically significant differences were found between the groups for these changes (p = 0.20, p = 0.53, p = 0.69, and p = 0.55, respectively).

**Fig. 4:**
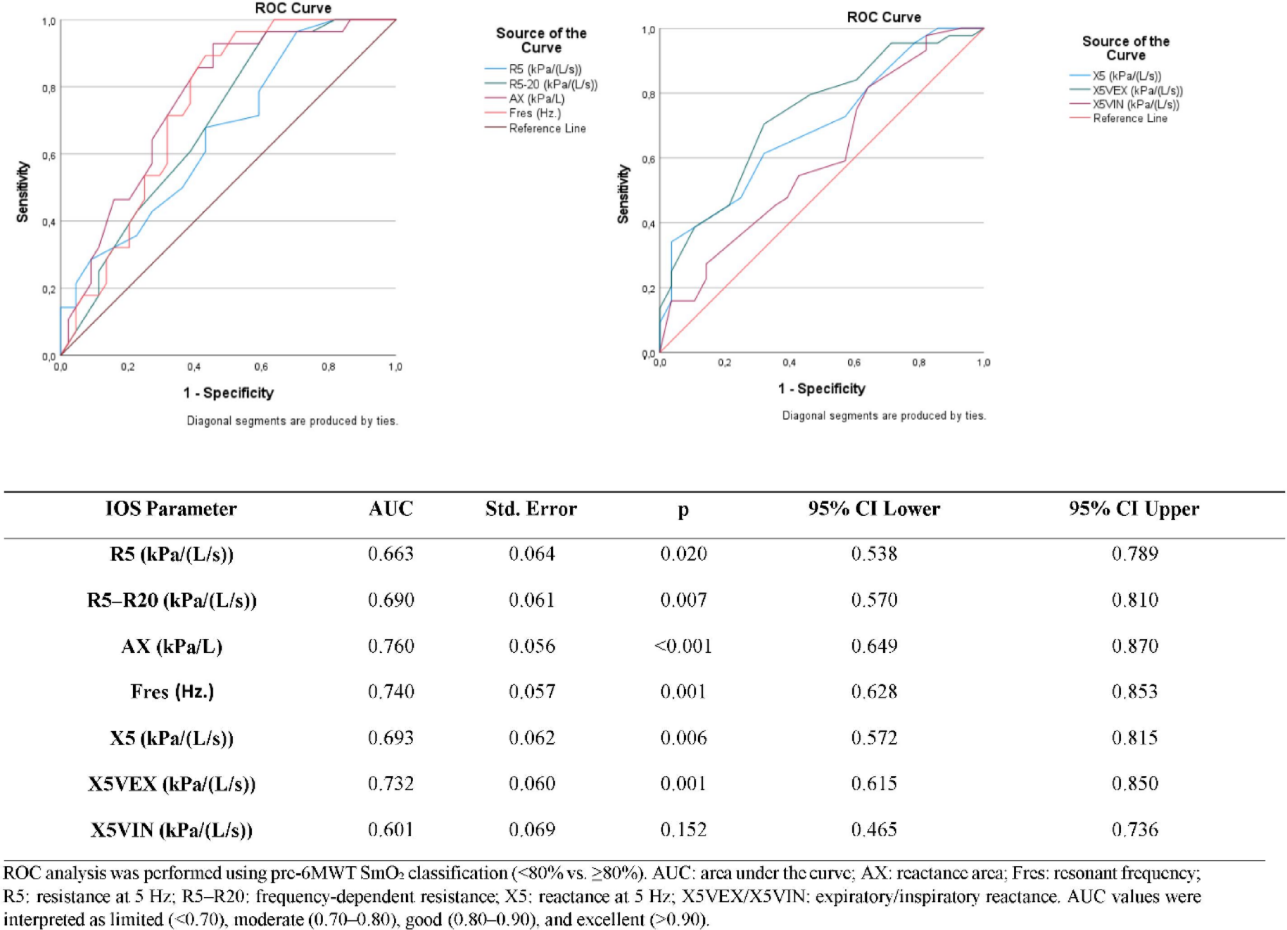
Receiver operating characteristic (ROC) analysis of pre-exercise IOS Parameters for identifying lower muscle oxygenation levels (pre-6MWT SmO_2_ < 80%) in the study population

ROC analysis was performed to evaluate whether pre-exercise IOS parameters could identify participants with lower SmO₂ levels (Fig. 4). Among the evaluated parameters, AX showed the highest discriminatory performance (AUC: 0.760, p < 0.001), followed by Fres (AUC: 0.740, p = 0.001) and X5VEX (AUC: 0.732, p = 0.001). R5, R5–R20, and X5 demonstrated limited-to-borderline discriminatory ability, whereas X5VIN did not show significant performance. Overall, IOS parameters demonstrated limited-to-moderate ability to identify lower muscle oxygenation levels.

## Discussion

In our study, although age, sex, FVC % predicted, FEV₁ % predicted, and FEV₁/FVC values were comparable between asymptomatic smokers and non-smokers, significant differences in respiratory system mechanics assessed by IOS were observed. In smokers, resistance components (R5, R10, R15, R20, R25, and R5–R20) were higher, while reactance components (X5, X5VEX, and X5VIN) were lower, with AX, Fres, and Z5 values also higher. These alterations were associated with smoking exposure, with correlation analyses showing positive associations for resistance parameters and inverse associations for reactance parameters. Regarding functional assessment, 6MWT distance was lower in smokers, and in the multivariable analysis, lower pre-6MWT R5 and higher X5VIN levels were associated with longer 6MWT distance. In addition, SmO₂ levels were lower in smokers both before and after exercise, and the between-group differences in IOS parameters persisted in the post-exercise assessment.

Previous studies suggest that small airway dysfunction may develop before the onset of classical spirometric impairment and that IOS may be sensitive in detecting these early changes. In asymptomatic smokers with preserved spirometry, peripheral airway indices, including R5–R20, AX, and Fres, have been shown to increase with smoking exposure, and these alterations may occur even when spirometric values remain within normal limits [3]. Similarly, in individuals with chronic respiratory symptoms and preserved spirometry, increases in IOS parameters have been reported to identify small airway dysfunction with greater sensitivity and to be associated with clinical symptoms [2]. Small airway dysfunction identified by IOS has also been shown to be associated with structural changes on computed tomography, such as emphysema and gas trapping [20], as well as with airway wall thickness measurements [9], supporting the notion that these parameters may reflect underlying structural alterations. Moreover, in smokers without airflow limitation, IOS-defined small airway dysfunction has been linked to impaired health-related quality of life, with R5 and X5 among the associated parameters [21]. Taken together, these findings suggest that IOS-detected changes are consistent with subclinical small airway involvement and may reveal physiologically relevant differences even in individuals with normal spirometry.

IOS changes observed in smokers are consistent with early mechanical heterogeneity in the small airways. Frequency-dependent dissociation in resistance (R5–R20) and changes in reactance parameters (more negative X5, increased AX and/or Fres) suggest heterogeneous ventilation distribution in the distal airways and the presence of regions with different time constants [5, 22]. This pattern is indicative of increased effective elastance. The simultaneous increase in resistance parameters and decrease in reactance parameters with smoking exposure further support this mechanical behavior. It should be noted that R5–R20 does not directly reflect a single anatomical region and may be influenced by upper airway effects and ventilation heterogeneity; therefore, its interpretation as a direct indicator of small airway caliber is limited [5, 22]. A shift of X5 toward more negative values, along with increases in AX and Fres, reflects mechanical changes consistent with increased system stiffness and ventilation heterogeneity [5, 22]. These alterations may become more pronounced during expiration. A more negative X5 during expiration has been associated with a tendency toward small airway collapse, while intrabreath reactance changes may provide additional information regarding airway closure and expiratory flow limitation [22, 23]. These mechanical changes in the small airways become more meaningful when considered alongside underlying structural remodeling. Airway wall thickening, inflammatory cell infiltration, and luminal mucus accumulation have been shown to disrupt ventilation distribution [24]. Because small airways contribute only minimally to total airway resistance, early pathological changes in this region may not be reflected in spirometric measurements for a prolonged period, a phenomenon often referred to as the “silent zone” [25]. The demonstrated relationship between small airway wall thickness and IOS parameters (particularly R5–R20 and Fres) supports the notion that the measured mechanical changes may correspond to underlying structural pathology [26]. Accordingly, IOS changes observed in smokers with preserved spirometry may represent a physiological manifestation of early small airway involvement [5, 22, 27].

The observation that IOS-detected alterations occur despite preserved percent-predicted spirometric values and FEV₁/FVC ratio indicates that these methods reflect different physiological aspects of airway function. While spirometry primarily reflects global airflow limitation during forced expiratory maneuvers, IOS provides a complementary perspective on airway mechanics by measuring frequency-dependent components of respiratory system impedance during tidal breathing [16, 22]. Therefore, direct concordance between the two methods should not be expected. The limited contribution of small airways to total airway resistance is considered one of the main reasons why early pathological changes may not be reflected in spirometric measurements [25]. In contrast, IOS parameters, due to their sensitivity to ventilation heterogeneity and peripheral airway mechanics, may reveal functional differences even when spirometry remains normal [22]. However, it should be acknowledged that indices such as R5–R20 do not directly represent a single anatomical structure and may be influenced by upper airway effects and ventilation heterogeneity [5, 22]. Comparative studies further support this distinction. In a study validated with endobronchial optical coherence tomography, oscillometric parameters were shown to be more sensitive than spirometric measurements in detecting early changes [26]. In larger cohort studies, substantial discordance has been reported between IOS and spirometry, indicating that these methods may exhibit varying sensitivity across different patient groups [28]. Taken together, these findings suggest that IOS should be considered not as an alternative to spirometry, but rather as a complementary assessment tool reflecting distinct physiological dimensions of airway function [22, 28].

The findings suggest that IOS-detected differences may reflect early physiological alterations in respiratory system mechanics, even in the absence of overt spirometric impairment. Although lower R5 and higher X5VIN were associated with longer 6MWT distance, this association should be interpreted cautiously and primarily as exploratory, given that 6MWT performance is influenced by multiple respiratory, peripheral, behavioral, and conditioning-related factors. This association may be partly related to the physiological characteristics of these IOS indices: R5 reflects total airway resistance, whereas X5VIN, obtained during the inspiratory phase, may provide a less variable estimate of peripheral airway mechanics. Oscillometric measurements are known to differ between inspiratory and expiratory phases, with the expiratory phase being more variable due to airway collapse and flow limitation; therefore, averaged values may obscure these differences [22, 23]. In this context, the fact that X5 represents an average across respiratory phases and that X5VEX may be influenced by increased variability during expiration may explain their weaker associations with functional capacity. Furthermore, between-group IOS differences were also observed after exercise, suggesting that these mechanical differences were not confined to the resting assessment. Lower muscle oxygenation levels observed before and after exercise in smokers may suggest that reduced exercise performance is not solely attributable to airway mechanics and may also involve peripheral or systemic factors. Studies in young smokers have demonstrated reduced muscle oxygenation during exercise, with this change being associated with smoking exposure [29]. Moreover, the reported relationship between muscle oxygenation and exercise capacity in patients with chronic obstructive pulmonary disease suggests that this physiological response may be observed across different clinical conditions [30]. Taken together, these findings suggest that IOS-detected mechanical differences may have potential functional relevance; however, this interpretation remains exploratory and should be considered in light of the multifactorial nature of exercise performance [27]. Given that IOS parameters are influenced by multiple physiological processes, these measurements should be interpreted in conjunction with clinical findings [22]. In addition, several IOS parameters demonstrated significant correlations with baseline SmO₂ levels, while ROC analysis showed limited-to-moderate discriminatory ability for identifying lower SmO₂ levels. These findings may indirectly reflect smoking-related ventilation heterogeneity and possible ventilation/perfusion imbalance; however, SmO₂ is also influenced by peripheral oxygen extraction, microvascular regulation, and muscle metabolic activity.

Nevertheless, although significant between-group differences were observed, several IOS values remained within conventionally accepted reference ranges. Therefore, these findings should be interpreted as subtle physiological alterations rather than definitive pathological airway dysfunction.

Our findings support the observations of Pisi et al., who showed that IOS can detect early small airway changes in asymptomatic smokers with preserved spirometry [3]. The present results further suggest that IOS may provide additional information when evaluated together with 6MWT performance and muscle oxygenation, particularly when measurements are obtained before and after exercise. The observed associations among IOS-detected differences, 6MWT performance, and muscle oxygenation suggest potential functional relevance; however, they do not establish a direct mechanistic relationship. Therefore, IOS parameters should be interpreted not in isolation but together with functional and clinical findings to allow a more comprehensive assessment of early physiological changes. This integrative approach supports the notion that smoking-related early involvement can be identified in a multidimensional manner before becoming clinically apparent.

The cross-sectional design limits the interpretation of the observed associations within a causal framework. The study population consisted of asymptomatic and relatively young individuals, which may limit the generalizability of the findings to older age groups or populations with clinically significant disease; however, this also allowed early-stage changes to be examined under conditions with fewer confounding factors. The relatively small sample size may have reduced the statistical power of some associations. IOS parameters are influenced by multiple physiological components and do not directly reflect a single anatomical region, which requires cautious interpretation of these measurements. The performance of measurements during tidal breathing and their sensitivity to technical factors represent additional limitations inherent to the nature of the method. 6MWT performance may also be affected by habitual physical activity, conditioning status, behavioral characteristics, and socioeconomic factors, which were not objectively quantified in this study. Future studies incorporating objective physical activity monitoring, such as actigraphy, may help clarify the contribution of these factors to exercise performance. Muscle oxygenation measurements may vary depending on local tissue characteristics and sensor placement.

## Conclusion

This study demonstrates that early alterations in respiratory system mechanics can be detected by IOS in asymptomatic smokers, even in the presence of preserved spirometric findings. The associations among IOS parameters, exercise capacity, and muscle oxygenation suggest that early small airway involvement may be functionally relevant, although exercise performance is influenced by multiple physiological and behavioral factors. These findings support the notion that the effects of smoking may emerge at multiple physiological levels in the early stages and may not be fully captured by conventional assessment methods alone. IOS may therefore serve as a complementary tool in the evaluation of this multidimensional involvement. Further studies including larger populations and longitudinal follow-up are needed to better clarify the clinical implications of these findings.

## Supplementary Information

Below is the link to the electronic supplementary material.

Supplementary Material 1

## Data Availability

The datasets generated and analyzed during the current study are available from the corresponding author upon reasonable request.

## Declarations

### Permission to Reproduce Material from Other Sources

No material from other sources requiring permission for reproduction was used in this manuscript.
