# Supplementary material for: Early Small Airway Mechanics and Functional Correlates in Asymptomatic Smokers: An Impulse Oscillometry-Based Study
Source: Lung. 2026 Jun 10;204(1):35. doi: 10.1007/s00408-026-00902-1 (PMC13249713; doi:10.1007/s00408-026-00902-1)
Supplement: Supplementary file 1 — Supplementary Material 1 [file 408_2026_902_MOESM1_ESM.docx]

**Supplementary Table 1.** Correlation analysis between pre-exercise IOS parameters and baseline muscle oxygen saturation (SmO₂)

| **Parameters** | **Correlation with baseline SmO₂ (r)** | **p-value** |
| --- | --- | --- |
| **R5 (kPa/(L/s))** | −0.272 | 0.021 |
| **R5–R20 (kPa/(L/s))** | −0.340 | 0.003 |
| **AX (kPa/L)** | −0.424 | <0.001 |
| **X5 (kPa/(L/s))** | 0.244 | 0.039 |
| **X5VEX (kPa/(L/s))** | 0.269 | 0.023 |
| **X5VIN (kPa/(L/s))** | 0.139 | 0.243 |
| **Fres (Hz)** | −0.398 | 0.001 |

Spearman correlation analysis between pre-exercise impulse oscillometry (IOS) parameters and baseline muscle oxygen saturation (SmO₂). AX: reactance area; Fres: resonant frequency; R5: resistance at 5 Hz; R5–R20: frequency-dependent resistance; SmO₂: muscle oxygen saturation; X5: reactance at 5 Hz; X5VEX/X5VIN: expiratory/inspiratory reactance.
